# Synthesis, Spectral Characterization, and Biological Evaluation of Transition Metal Complexes of Bidentate N, O Donor Schiff Bases

**DOI:** 10.1155/2014/812924

**Published:** 2014-07-23

**Authors:** Sajjad Hussain Sumrra, Muhammad Ibrahim, Sabahat Ambreen, Muhammad Imran, Muhammad Danish, Fouzia Sultana Rehmani

**Affiliations:** ^1^Department of Chemistry, Institute of Chemical and Biological Sciences, University of Gujrat, Gujrat 50700, Pakistan; ^2^Department of Applied Chemistry, Government College University, Faisalabad 38000, Pakistan; ^3^Department of Chemistry, University of Karachi, Karachi 75270, Pakistan; ^4^Department of Chemistry, Government Emerson College, Multan 60700, Pakistan

## Abstract

New series of three bidentate N, O donor type Schiff bases **(L** ^**1**^ **)**–**(L** ^**3**^ **)** were prepared by using ethylene-1,2-diamine with 5-methyl furfural, 2-anisaldehyde, and 2-hydroxybenzaldehyde in an equimolar ratio. These ligands were further complexed with Co(II), Cu(II), Ni(II), and Zn(II) metals to produce their new metal complexes having an octahedral geometry. These compounds were characterized on the basis of their physical, spectral, and analytical data. Elemental analysis and spectral data of the uncomplexed ligands and their metal(II) complexes were found to be in good agreement with their structures, indicating high purity of all the compounds. All ligands and their metal complexes were screened for antimicrobial activity. The results of antimicrobial activity indicated that metal complexes have significantly higher activity than corresponding ligands. This higher activity might be due to chelation process which reduces the polarity of metal ion by coordinating with ligands.

## 1. Introduction 

**Figure 1:**
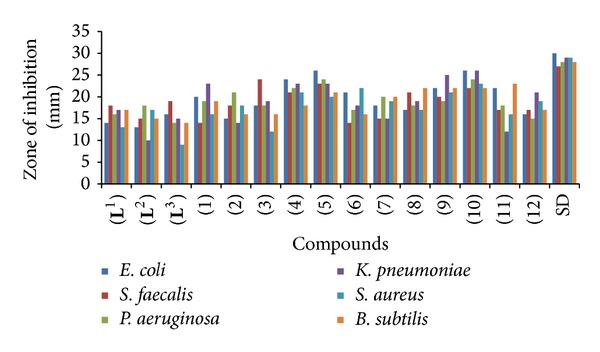
Comparison of antibacterial activity of Schiff bases versus metal(II) complexes.

**Figure 2:**
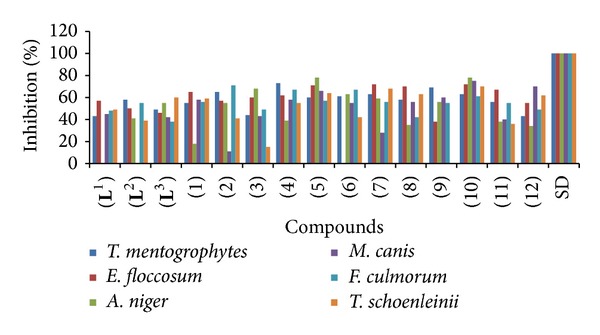
Comparison of antifungal activity of Schiff bases versus metal(II) complexes.

Schiff bases played an important role as ligands even a century after their discovery in coordination chemistry [1]. Schiff bases are derived from the condensation reaction of aromatic/aliphatic aldehydes and amines. They are an important class of organic ligands being extensively studied. Schiff base complexes of transition metals are still relevant to be of great interest in inorganic chemistry, although this topic has been extensively studied [2–4]. The chelating ability and biological applications of metal complexes have attracted remarkable attention [5]. Metal complexes having N, O donor atoms are very important because of their significant biological properties such as antibacterial [6, 7], antifungal [8], anticancer [9], and herbicidal [10] activity. In view of the significant structural and biological applications of ethylenediamine compounds, we wish to report the synthesis of a new class of Schiff bases** (L** ^**1**^ **)**–**(L** ^**3**^ **)**, derived from the reaction of ethylene-1,2-diamine with 5-methyl furfural, 2-anisaldehyde, and 2-hydroxybenzaldehyde, respectively, and their Co(II), Cu(II), Ni(II), and Zn(II) metal complexes **(1)**–**(12)** (Scheme 2). The compounds were characterized on the basis of physical properties, elemental analysis, infrared and UV-visible spectra, and antimicrobial activities. The Schiff bases and their metal chelates were screened for antibacterial activity against six bacterial strains:* Escherichia coli*,* Streptococcus faecalis*,* Pseudomonas aeruginosa*,* Klebsiella pneumoniae*,* Staphylococcus aureus,* and* Bacillus subtilis,* and also screened for antifungal activity against following six fungal strains:* Trichophyton mentogrophytes*,* Epidermophyton floccosum*,* Aspergillus niger*,* Microsporum canis*,* Fusarium culmorum,* and* Trichophyton schoenleinii.* The Schiff bases showed increased antibacterial activity against certain strains and their activities were enhanced on chelation (see Figures 1 and 2).

## 2. Experimental

### 2.1. Materials and Methods

Chemicals used were of analytical grade and purchased from commercial sources Sigma Aldrich and were used without further purification. All ligand synthesis reactions were carried out in solvents that were purified and dried before use, using standard literature methods. The redistilled and deionized water was used in all experiments. Gallenkamp apparatus was used to determine melting points of synthesized ligands and decomposition temperature of the metal complexes. Infrared spectra of solids (in a KBr matrix) were recorded in the 3700–370 cm^−1^ region on a Nicolet FT-IR Impact 400D infrared spectrometer. ^1^H and ^13^CNMR spectra were run on a Bruker Advance 300 MHz instrument. Mass spectrometry work was carried out by Ms. B. Woods N.U.I. Maynooth using an Agilent Technologies 6210 Time-of-Flight LC/MS. UV spectra were obtained on a Hitachi UV-3200 spectrophotometer. Microanalysis (C, H, and N%) of the synthesized compounds was carried out using a CHN Analyzer on Perkin Elmer 2400 series II. Molar conductances of the transition metal complexes were measured in 0.01 M in DMF solution using an Inolab Cond 720 Conductivity Bridge at room temperature. A Stanton SM12/S Gouy balance was used to measure the magnetic susceptibility of the metal complexes at room temperature by using mercury acetate as a standard.

### 2.2. Chemistry of Synthesis of Ligands

Different aldehydes such as 5-methyl furfural, 2-anisaldehyde, and 2-hydroxybenzaldehyde in methanol (20 mL) were added to a refluxed solution of ethylene-1,2-diamine in same solvent in an equimolar ratio for 10 minutes followed by 2-3 drops of acetic acid. Then the reaction mixture was refluxed for 6 h by monitoring through TLC. When the reaction was completed, it was cooled to room temperature, filtered, and volume reduced to about one-third using rotary evaporator. The solid product thus obtained was filtered, washed with methanol, and dried. It was recrystallized in hot methanol/ether (2 : 1). The ligands** (L** ^**1**^ **)**–**(L** ^**3**^ **)** were prepared by following the above mentioned method.

#### 2.2.1. N-[(E)-(5-Methylfuran-2-yl)methylidene]ethane-1,2-diamine (**L** ^**1**^)

Yield (1.12 g, 73%), mp 175°C; color reddish brown. ^1^H NMR (ppm d_6_-DMSO) 2.35 (s, CH_3_), 3.05 (s, 2H), 3.68 (s, 2H), 4.85 (s, NH_2_), 6.34 (d, 2H), 6.68 (d, 2H), 7.18 (s, HC=N); ^13^C NMR: (ppm d_6_-DMSO): 13.5, 43.0, 55.8, 107.2, 116.6, 146.4, 153.3, 162.6; IR (KBr, cm^−1^): 3250 (NH_2_), 1632 (HC=N), 1575, 1545 (C=C), 1090 (C–O); Mass Spectrum (ESI) [M]^+^ = 152.19. Anal. calcd. for C_8_H_12_N_2_O (152.19): C, 63.13; H, 7.95; N, 18.41. Found: C, 63.08; H, 7.93; N, 18.37.

#### 2.2.2. N-[(E)-(2-Methoxyphenyl)methylidene]ethane-1,2-diamine (**L** ^**2**^)

Yield (1.21 g, 67%); mp 142°C; color dark brown. ^1^H NMR (ppm d_6_-DMSO): 2.95 (s, OCH_3_), 3.15 (s, 2H), 3.85 (s, 2H), 4.87 (s, NH_2_), 6.87 (t, 1H), 6.95 (d, 1H), 7.32 (t, 1H), 7.59 (d, 1H), 8.78 (s, HC=N); ^13^C NMR: (ppm d_6_-DMSO): 44.2, 53.6, 56.3, 114.7, 122.9, 123.7, 129.6, 134.5, 160.5, 162.3; IR (KBr, cm^−1^): 3255 (NH_2_), 2920 (OCH_3_), 1635 (HC=N), 1577, 1543 (C=C); Mass Spectrum (ESI): [M]^+^ = 178.23. Anal. calcd. for C_10_H_14_N_2_O (178.23): C, 67.39; H, 7.92; N, 15.72. Found: C, 67.33; H, 7.88; N, 15.69.

#### 2.2.3. 2-{(E)-[(2-Aminoethyl)imino]methyl}phenol (**L** ^**3**^)

Yield (1.20 g, 73%); mp: 155°C, color (yellow). ^1^H NMR (ppm d_6_-DMSO): 3.40 (s, 2H), 3.98 (s, 2H), 4.89 (s, NH_2_), 6.93 (t, 1H), 7.11 (d, 1H), 7.43 (t, 1H), 7.72 (d, 1H), 8.85 (s, HC=N), 9.97 (s, OH); ^13^C NMR (ppm d_6_-DMSO): 44.8, 52.9, 118.4, 119.5, 122.2, 131.5, 133.1, 159.2, 162.3; IR (KBr, cm^−1^): 3385 (OH), 3253 (NH_2_), 1638 (HC=N); Mass Spectrum (ESI): [M]^+^ = 164.20. Anal. calcd. for C_9_H_12_N_2_O (164.20): C, 65.83; H, 7.37; N, 17.06. Found: C, 65.77; H, 7.32; N, 17.02.

### 2.3. Chemistry of Synthesis of the Transition Metal(II) Complexes

All complexes were prepared according to the following procedure: to a hot magnetically refluxed methanol solution (30 mL) of the respective Schiff base ligand (10 mmol), a methanol solution (20 mL) of respective metal(II) salt chloride·*n*H_2_O (5 mmol) was added (*n* = 0, 2 or 6). The mixture was refluxed for 3 h, during which a precipitated product was formed. It was then cooled to room temperature, filtered, and washed with methanol and finally with diethyl ether. The precipitated product thus obtained was dried and recrystallized in a mixture of hot aqueous methanol (1 : 2) to obtain TLC pure product.

#### 2.3.1. Co(II) Metal Complex of (**L** ^**1**^) (**1**)

Yield (1.45 g, 62%), mp 232–234°C, IR (KBr) 3465 (H_2_O), 1617 (HC=N), 1074 (C–O), 525 (M–N), 457 (M–O); UV (DMSO) *λ*_max⁡_ (cm^−1^) 8515, 17511 and 29982; conductance (Ω^−1^ cm^2^ mol^−1^) 98.5; B.M. (*μ*_eff_): 4.68. Anal. calcd. for C_16_H_28_N_4_O_4_CoCl_2_ (470.35); C, 40.78; H, 5.95; N, 11.91; Co, 12.53. Found: C, 40.71; H, 5.91; N, 11.88; Co, 12.48.

#### 2.3.2. Ni(II) Metal Complex of (**L** ^**1**^) (**2**)

Yield (1.50 g, 64%), mp 225–227°C; IR (KBr): 3474 (H_2_O), 1619 (HC=N), 1077 (C–O), 527 (M–N), 454 (M–O); UV (DMSO) *λ*_max⁡_ (cm^−1^): 8623, 17620, 25890 and 29895; conductance (Ω^−1^ cm^2^ mol^−1^): 97.4; B.M. (*μ*_eff_): 3.42. Anal. calcd. for C_16_H_28_N_4_O_4_NiCl_2_ (470.11); C, 40.84; H, 5.95; N, 11.91, Ni, 12.48. Found: C, 40.77; H, 5.93; N, 11.86; Ni, 12.45.

#### 2.3.3. Cu(II) Metal Complex of (**L** ^**1**^) (**3**)

Yield (1.37 g, 58%); mp 238–240°C; IR (KBr): 3469 (H_2_O), 1616 (HC=N), 1072 (C–O), 523 (M–N), 454 (M–O); UV (DMSO) *λ*_max⁡_ (cm^−1^): 8515, 17511 and 29982; conductance (Ω^−1^ cm^2^ mol^−1^): 98.9; B.M. (*μ*_eff_): 1.97. Anal. calcd. for C_16_H_28_N_4_O_4_CuCl_2_ (474.96); C, 40.42; H, 5.89; N, 11.79; Cu, 13.38. Found: C, 40.34; H, 5.83; N, 11.72; Cu, 13.31.

#### 2.3.4. Zn(II) Metal Complex of (**L** ^**1**^) (**4**)

Yield (1.51 g, 63%); mp 216–218°C; ^1^H NMR: (ppm d_6_-DMSO): 2.46 (s, CH_3_), 3.15 (s, 2H), 3.83 (s, 2H), 4.98 (s, NH_2_), 6.48 (d, 2H), 6.80 (d, 2H), 7.48 (s, HC=N), 10.5 (s, 4H, H_2_O); ^13^C NMR (ppm d_6_-DMSO): 13.9, 43.4, 56.4, 107.9, 117.7, 147.6, 154.6, 163.8; IR (KBr): 3480 (H_2_O), 1612 (HC=N), 1077 (C–O), 529 (M–N), 459 (M–O); UV (DMSO) *λ*_max⁡_ (cm^−1^): 28382; conductance (Ω^−1^ cm^2^ mol^−1^): 97.5; B.M. (*μ*_eff_): diamagnetic. Anal. calcd. for C_16_H_28_N_4_O_4_ZnCl_2_ (476.82); C, 40.26; H, 5.87; N, 11.74; Zn, 13.71. Found: C, 40.18; H, 5.83; N, 11.69; Zn, 13.66.

#### 2.3.5. Co(II) Metal Complex of (**L** ^**2**^) (**5**)

Yield (1.55 g, 59%), mp 248–251°C, IR (KBr) 3465 (H_2_O), 1621 (HC=N), 1380 (C–O), 532 (M–N), 465 (M–O); UV (DMSO) *λ*_max⁡_ (cm^−1^) 8690, 17823 and 29622; conductance (Ω^−1^ cm^2^ mol^−1^) 97.7; B.M. (*μ*_eff_): 4.55. Anal. calcd. for C_20_H_32_N_4_O_4_CoCl_2_ (522.42); C, 45.94; H, 6.12; N, 10.71; Co, 11.28. Found: C, 45.88; H, 6.08; N, 11.69; Co, 11.28.

#### 2.3.6. Ni(II) Metal Complex of (**L** ^**2**^) (**6**)

Yield (1.58 g, 61%), mp 229–231°C; IR (KBr): 3474 (H_2_O), 1617 (HC=N), 1383 (C–O), 520 (M–N), 447 (M–O); UV (DMSO) *λ*_max⁡_ (cm^−1^): 8710, 17850, 25715 and 29675; conductance (Ω^−1^ cm^2^ mol^−1^): 98.2; B.M. (*μ*_eff_): 3.55. Anal. calcd. for C_20_H_32_N_4_O_4_NiCl_2_ (522.18); C, 45.99; H, 6.12; N, 10.71, Ni, 11.23. Found: C, 45.92; H, 6.09; N, 11.66; Ni, 11.28.

#### 2.3.7. Cu(II) Metal Complex of (**L** ^**2**^) (**7**)

Yield (1.71 g, 65%); mp 252–255°C; IR (KBr): 3469 (H_2_O), 1615 (HC=N), 1377 (C–O), 535 (M–N), 459 (M–O); UV (DMSO) *λ*_max⁡_ (cm^−1^): 8705, 17215 and 29528; conductance (Ω^−1^ cm^2^ mol^−1^): 97.7; B.M. (*μ*_eff_): 1.92. Anal. calcd. for C_20_H_32_N_4_O_4_CuCl_2_ (527.04); C, 45.53; H, 6.07; N, 10.62; Cu, 12.05. Found: C, 45.48; H, 6.01; N, 10.69; Cu, 12.01.

#### 2.3.8. Zn(II) Metal Complex of (**L** ^**2**^) (**8**)

Yield (1.74 g, 66%); mp 259–262°C; ^1^H NMR: (ppm d_6_-DMSO): 3.03 (s, OCH_3_), 3.22 (s, 2H), 3.93 (s, 2H), 4.96 (s, NH_2_), 6.95 (t, 1H), 7.02 (d, 1H), 7.42 (t, 1H), 7.65 (d, 1H), 8.93 (s, HC=N), 10.5 (s, 4H, H_2_O); ^13^C NMR (ppm d_6_-DMSO): 44.6, 53.9, 56.8, 115.3, 123.7, 124.6, 130.4, 134.9, 160.8, 163.5; IR (KBr): 3480 (H_2_O), 1612 (HC=N), 1382 (C–O), 527 (M–N), 454 (M–O); UV (DMSO) *λ*_max⁡_ (cm^−1^): 28538; conductance (Ω^−1^ cm^2^ mol^−1^): 93.5; B.M. (*μ*_eff_): diamagnetic. Anal. calcd. for C_20_H_32_N_4_O_4_ZnCl_2_ (528.90); C, 45.37; H, 6.05; N, 10.58; Zn, 12.36. Found: C, 45.28; H, 5.99; N, 11.51; Zn, 12.28.

#### 2.3.9. Co(II) Metal Complex of (**L** ^**3**^) (**9**)

Yield (1.41 g, 67%), mp 224–227°C, IR (KBr) 3465 (H_2_O), 1623 (HC=N), 1377 (C–O), 538 (M–N), 455 (M–O); UV (DMSO) *λ*_max⁡_ (cm^−1^) 8587, 17967 and 29745; conductance (Ω^−1^ cm^2^ mol^−1^) 15.6; B.M. (*μ*_eff_): 4.32. Anal. calcd. for C_18_H_26_N_4_O_4_Co (421.36); C, 51.31; H, 6.22; N, 13.30; Co, 13.99. Found: C, 51.22; H, 6.16; N, 13.24; Co, 13.92.

#### 2.3.10. Ni(II) Metal Complex of (**L** ^**3**^) (**10**)

Yield (1.58 g, 61%), mp 229–231°C; IR (KBr): 3474 (H_2_O), 1619 (HC=N), 1381 (C–O), 535 (M–N), 441 (M–O); UV (DMSO) *λ*_max⁡_ (cm^−1^): 8599, 17645, 25661 and 29717; conductance (Ω^−1^ cm^2^ mol^−1^): 14.2; B.M. (*μ*_eff_): 3.39. Anal. calcd. for C_18_H_26_N_4_O_4_Ni (421.12); C, 51.34; H, 6.22; N, 13.30, Ni, 13.94. Found: C, 51.26; H, 6.19; N, 13.26; Ni, 13.88.

#### 2.3.11. Cu(II) Metal Complex of (**L** ^**3**^) (**11**)

Yield (1.34 g, 63%); mp 235–237°C; IR (KBr): 3469 (H_2_O), 1619 (HC=N), 1375 (C–O), 539 (M–N), 448 (M–O); UV (DMSO) *λ*_max⁡_ (cm^−1^): 8670, 17371 and 29732; conductance (Ω^−1^ cm^2^ mol^−1^): 13.4; B.M. (*μ*_eff_): 1.93. Anal. calcd. for C_18_H_26_N_4_O_4_Cu (425.96); C, 50.75; H, 6.15; N, 13.15; Cu, 14.92. Found: C, 50.68; H, 6.11; N, 13.10; Cu, 14.85.

#### 2.3.12. Zn(II) Metal Complex of (**L** ^**3**^) (**12**)

Yield (1.51 g, 71%); mp 239–241°C; ^1^H NMR: (ppm d_6_-DMSO): 3.54 (s, 2H), 4.14 (s, 2H), 4.0 (s, NH_2_), 7.09 (t, 1H), 7.34 (d, 1H), 7.55 (t, 1H), 7.87 (d, 1H) 8.98 (s, HC=N), 10.5 (s, 4H, H_2_O); ^13^C NMR (ppm d_6_-DMSO): 45.2, 53.5, 118.7, 119.7, 122.7, 131.9, 133.5, 159.7, 162.9; IR (KBr): 3480 (H_2_O), 1619 (HC=N), 1376 (C–O), 532 (M–N), 445 (M–O); UV (DMSO) *λ*_max⁡_ (cm^−1^): 28653; conductance (Ω^−1^ cm^2^ mol^−1^): 13.5; B.M. (*μ*_eff_): diamagnetic. Anal. calcd. for C_18_H_26_N_4_O_4_Zn (427.83); C, 50.53; H, 6.13; N, 13.10; Zn, 15.29. Found: C, 50.45; H, 6.09; N, 13.05; Zn, 15.22.

### 2.4. Biological Activity

#### 2.4.1. *In Vitro* Antibacterial Activity

All newly synthesized Schiff bases** (L** ^**1**^ **)**–**(L** ^**3**^ **)** and their transition metal(II) complexes** (1)**–**(12)** were screened for their* in vitro* antibacterial activity against (*Escherichia coli*,* Streptococcus faecalis*,* Pseudomonas aeruginosa*,* Klebsiella pneumoniae*,* Staphylococcus aureus,* and* Bacillus subtilis*) bacterial strains by the agar-well diffusion method [11] and recorded in Table 1. Small portion (10 mL) of nutrient broth was inoculated with the test organisms and incubated at 37°C for 24 h. Using a sterile pipette, 0.6 mL of the broth culture of the test organism was added to 60 mL of molten agar which had been cooled to 45°C, mixed well, and poured into a sterile petri dish. Duplicate plates of each organism were prepared. The agar was allowed to set and harden and the required numbers of holes were cut using a sterile cork borer ensuring proper distribution of holes on the border and one in the center. Agar plugs were removed. Different cork borers were used for different test organisms. Using a 0.1 mL pipette, 100 *μ*L of the test sample dissolved in an appropriate solvent was poured into appropriately labelled cups. The same concentrations of the standard antibacterial agent (streptomycin in 1 mg/mL) and the solvent (as control) were used. The plates were left at room temperature for 2 h to allow diffusion of the sample and incubated face upwards at 37°C for 24 h. The diameter of the zones of inhibition was measured to the nearest mm.

#### 2.4.2. *In Vitro* Antifungal Activity

Antifungal activities of all compounds were studied against six fungal strains* Trichophyton mentogrophytes*,* Epidermophyton floccosum*,* Aspergillus niger*,* Microsporum canis*,* Fusarium culmorum,* and* Trichophyton schoenleinii* according to recommended procedure [12] and recorded in Table 2. Test sample was dissolved in sterile DMSO to serve as stock solution. Sabouraud dextrose agar was prepared by mixing Sabouraud 4% glucose agar and agar in distilled water. It was then stirred with a magnetic stirrer to dissolve it and a known amount was dispensed into screw capped test tubes. Test tubes containing media were autoclaved at 121°C for 15 min. Tubes were allowed to cool to 50°C and the test sample of desired concentrations pipetted from the stock solution into the nonsolidified Sabouraud agar media. Tubes were then allowed to solidify in a slanting position at room temperature. Each tube was inoculated with a 4 mm diameter piece of inoculum removed from a seven-day-old culture of fungi.

#### 2.4.3. Minimum Inhibitory Concentration (MIC)

Compounds containing promising antibacterial activity were selected for minimum inhibitory concentration (MIC) studies [13]. The minimum inhibitory concentration was determined using the disc diffusion technique by preparing discs containing 10, 25, 50, and 100 *μ*g mL^−1^ concentrations of the compounds along with standards at the same concentrations.

## 3. Results and Discussion

**Scheme 1:**
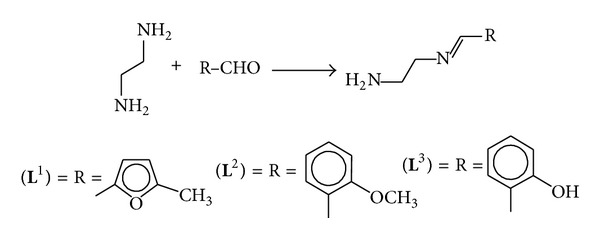


The condensation of ethylene-1,2-diamine and 5-methyl furfural, 2-anisaldehyde, and 2-hydroxybenzaldehyde in 1 : 1 molar ratio afforded three Schiff base ligands** (L** ^**1**^ **)**–**(L** ^**3**^ **)** (Scheme 1). These ligands were air and moisture stable compounds. All of them were colored compounds. These were microcrystalline solids which melted at 145–175°C. All were soluble in DMSO and DMF at room temperature and soluble on heating in methanol and ethanol.

**Scheme 2:**
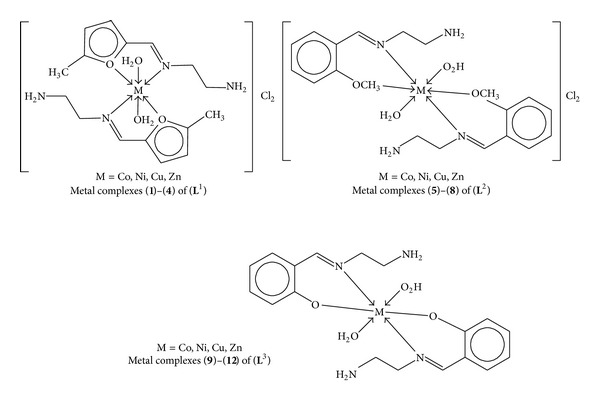


These bidentate ligands reacted readily with Co(II), Cu(II), Ni(II), and Zn(II) metals as their chlorides [CoCl_2_·6H_2_O, NiCl_2_·6H_2_O, CuCl_2_·2H_2_O, and ZnCl_2_] in methanol to form their metal(II) complexes (Scheme 2). All the synthesized metal(II) complexes were intensely colored except Zn(II) complexes which were white and all complexes were microcrystalline in nature. The metal(II) complexes decomposed without melting. They were all insoluble in common organic solvents such as ethanol, methanol, dichloromethane, and acetone but soluble in DMSO and DMF.

The spectral data and elemental analysis of the prepared ligands and their metal(II) complexes were in good agreement with their structure, indicating the high purity of all the compounds. The analytical data of the complexes indicated a 1 : 2 metal : ligand stoichiometry.

### 3.1. IR Spectra

These ligands can coordinate through the azomethine-N, furanyl-O, methoxy-O, and oxygen atom from the deprotonation of the phenolic group. Some of the characteristic IR spectral data were reported in experimental part. The ligands** (L** ^**1**^ **)**–**(L** ^**3**^ **)** displayed band at 3250–3255 cm^−1^ resulting from NH_2_ vibrations [14]. The ligand** (L** ^**3**^ **)** showed band resulting from OH vibrations [15] at 3385 cm^−1^. However, the IR spectra of the ligand** (L** ^**2**^ **)** demonstrated vibrations at 2920 cm^−1^ due to OCH_3_ stretching [16]. The Schiff bases** (L** ^**1**^ **)**–**(L** ^**3**^ **)** possessed the characteristic azomethine (HC=N) stretching [17] at 1632–1638 cm^−1^, hence giving clue of condensation product. The ligand** (L** ^**1**^ **)** showed the bands at 1090 cm^−1^ due to (C–O) vibrations [18]. The comparison of the IR spectra of the Schiff bases** (L** ^**1**^ **)**–**(L** ^**3**^ **)** with their metal(II) complexes** (1)**–**(12)** indicated that the Schiff bases were principally coordinated to the metal(II) ions bidentately. The IR bands of azomethine group appearing in Schiff bases complexes shifted to lower frequency (10–15 cm^−1^) at 1612–1623 cm^−1^ confirming the coordination of the azomethine nitrogen [19] with the metal(II) atoms. IR bands at 3250–3255 cm^−1^ resulting from NH_2_ vibrations of ligands** (L** ^**1**^ **)**–**(L** ^**3**^ **)** remained unchanged in all the complexes showing their no involvement in the coordination. The following evidences further support the mode of chelation.

- Appearance of the new bands in their metal complexes at 520–539 and 441–465 cm^−1^ which were assigned to* v*(M–N) [20] and* v*(M–O) vibrations, respectively, and these bands were absent in their uncomplexed ligands.
- The (C–O) vibrations of ligand** (L** ^**1**^ **)** at 1090 cm^−1^ were shifted to lower frequency 1072–1077 cm^−1^ in the metal(II) complexes** (1)**–**(4)**. This, in turn, supported the evidence of the participation of heteroatom-O in the coordination.
- Appearance of the new bands at 1377–1383 cm^−1^ due to* v*(C–O) vibrations in the metal(II) complexes** (5)**–**(8)** indicated the coordination of OCH_3_ group with the metal atoms [21].
- The disappearance of *ν*(OH) band at 3385 cm^−1^ in** (8)**–**(12)** complexes and appearance of new bands at 1375–1381 cm^−1^ due to the *ν*(C–O) stretching mode in the complexes revealed the deprotonation of the hydroxyl OH group found in the ligand** (L** ^**3**^ **)**. It, in turn, indicated that the proton of the OH group was replaced by the metal ions in the formation of complexes.
- All the metal(II) complexes displayed new broad peaks at 3465–3480 cm^−1^ which were assigned to water molecules.

These new bands were only observed in the spectra of the complexes but absent in the spectra of the Schiff bases. Therefore, these clues supported the evidence of the participation of heteroatom-O, deprotonation of benzilidene-O, and azomethine-N in the coordination. All these evidences compromise with the complexation of the metal(II) ions to the prepared Schiff bases.

### 3.2. ^1^H NMR Spectra

^1^H NMR spectra of the Schiff bases and their diamagnetic Zn(II) complexes were recorded in DMSO-d_6_. ^1^H NMR spectral data of the Schiff bases** (L** ^**1**^ **)**–(**L** ^**3**^ **)** and their diamagnetic Zn(II) complexes are provided in the experimental section. The ^1^H NMR spectra of the Schiff base ligands** (L** ^**1**^ **)**–**(L** ^**3**^ **)** demonstrated characteristic amino (N**H**_**2**_) and azomethine (C**H**=N) protons at 4.85–4.89 and 7.18–8.85 ppm as a singlet, respectively. The (CH_3_) protons of the ligands** (L** ^**1**^ **)** were observed at 2.35 ppm as a singlet. The (OC**H**_**3**_) proton present in the ligand** (L** ^**2**^ **)** was observed at 2.95 ppm as a singlet. The (C**H**_**2**_) protons present in all the ligands** (L** ^**1**^ **)**–**(L** ^**3**^ **)** were observed at 3.05–3.98 ppm as a singlet. In case of the ligand** (L** ^**3**^ **)** the O–**H** proton was observed at 9.97 ppm as a singlet. The furan protons of ligand** (L** ^**1**^ **)** were found at 6.34–6.68 ppm as a doublet. The phenyl protons found in ligands** (L** ^**2**^ **)** and** (L** ^**3**^ **)** were found at 6.87–7.75 ppm as a doublet, double doublet, and triplet.

The coordination of the azomethine (HC=N) nitrogen was assigned by the downfield shifting of the azomethine proton signal from 7.18–8.85 in fee ligands to 8.78–8.88 ppm in their Zn(II) complexes, respectively. This downfield shifting of azomethine proton in Zn(II) complexes was attributed to the discharging of electronic cloud towards the Zn(II) ion. The hydroxyl (OH) proton at 9.97 ppm in the ligand (**L** ^**3**^) disappeared in the spectra of its Zn(II) complexes, indicating deprotonation and coordination of the oxygen with the metal ion. All other protons underwent downfield shift by 0.7–0.30 ppm owing to the increased conjugation on complexation with the zinc metal atom. Thus, the number of protons calculated from the integration curves [22, 23] and obtained values of the expected CHN analysis agreed well with each other.

### 3.3. ^13^C NMR Spectra

^13^C NMR spectra of the Schiff bases and their diamagnetic Zn(II) complexes were recorded in DMSO-d_6_. The ^13^C NMR spectral data are reported along with their possible assignments in the Experimental section and all the carbons were found in the expected regions. The ^13^CNMR spectra of the Schiff base ligands** (L** ^**1**^ **)**–**(L** ^**3**^ **)** showed characteristic azomethine (CH=N) carbons at 161.7–163.9 ppm. The (CH_3_), (CH_2_), and (OCH_3_) carbons of the ligands were observed at 13.5, 43.0–55.8, and 56.3 ppm, respectively. All the furanyl and phenyl carbons were found at 107.2–160.5 ppm.

Downfield shifting of the azomethine carbons from *δ* 161.7–163.9 ppm in the free ligands to 162.9–163.8 ppm in its Zn(II) complexes was due to shifting of electronic density towards the Zn(II) ion. Similarly, all carbons of hetero-aromatic and phenyl rings being near to the coordination sites also showed downfield shifting by 0.10–0.60 ppm due to the increased conjugation and coordination with the metal atoms. The downfield shifting also confirmed the coordination of the azomethine to the zinc metal atom. Moreover, the presence of the number of carbons is well in agreement with the expected values [24, 25]. Furthermore, the conclusions drawn from these studies present further support to the modes of bonding discussed in their IR and ^1^H NMR spectra.

### 3.4. Mass Spectra

The mass fragmentation pattern of the ligands** (L** ^**1**^ **)**–**(L** ^**3**^ **)** followed the cleavage of C=N (exocyclic), C=C, and C–O bonds. The mass spectral data and the most stable fragmentation values of the ligands were depicted in Experimental section. All the ligands showed pronounced molecular ion peaks. The data of the Schiff bases shown by mass spectra strongly confirmed the formation of the ligands possessing proposed structures and also their bonding pattern.

### 3.5. Molar Conductances

Molar conductance studies of the complexes were carried out in DMF. The data of molar conductances (93.5–98.7 ohm^−1^ cm^2^ mol^−1^) of metal(II) complexes** (1)**–**(8)** showed that these complexes were electrolytic [26] in nature. The metal(II) complexes** (9)**–**(12)** exhibited conductances in the range 13.1–15.9, thus indicating their nonelectrolytic [27, 28] nature.

### 3.6. Magnetic Measurements

The magnetic moment (B.M) values of all the metal(II) complexes,** (1)**–**(12),** were obtained at room temperature. The observed magnetic moment values of Co(II) complexes were found in the range of 4.32–4.68 B.M indicating the Co(II) complexes as high-spin suggesting three unpaired electrons in an octahedral environment [29]. The Ni(II) complexes showed magnetic moment values in the range of 3.39–3.55 B.M indicative of two unpaired electrons per Ni(II) ion suggesting these complexes to have an octahedral [30] geometry. The measured magnetic moment values 1.93–1.97 B.M for Cu(II) complexes are indicative of one unpaired electron per Cu(II) ion for d^9^-system suggesting octahedral [31] geometry. All the Zn(II) complexes were found to be diamagnetic [32] as expected.

### 3.7. Electronic Spectra

The electronic spectra of Co(II) complexes generally exhibited [33] three absorption bands in the regions 8515–8690, 17511–17967, and 29542–29982 cm^−1^ which may be assigned to 4T_1_g→4T_2_g(F), 4T_1_g→4A_2_g(F), and 4T_1_g→4Tg(P) transitions, respectively, and are suggestive of octahedral geometry around the Co(II) ion. The electronic spectral data of Ni(II) complexes showed [34] the bands in the regions 8599–8762, 17620–17850, and 25661–25890 cm^−1^ assigned, respectively, to the d-d transitions of 3A_2_g(F)→3T_2_g(F) and 3A_2_g(F)→3T_1_g(F). Also a strong band due to metal to ligand charge transfer appeared at 29675–29895 cm^−1^. The electronic spectra of all the Cu(II) complexes exhibited [35] absorption bands in the region at 8515–8737 and 17215–17672 cm^−1^ which may be assigned to the transitions 2Eg→2T_2_g. The high energy band at 29528–29982 cm^−1^ was due to forbidden ligand to metal charge transfer. On the basis of electronic spectra, octahedral geometry around the Cu(II) ion was suggested. The Zn(II) complexes did not show any d-d transition thus showing diamagnetic nature and their spectra were dominated only by a charge transfer band [36] at 28382–28653 cm^−1^.

### 3.8. Biological Evaluation

#### 3.8.1. Antibacterial Bioassay (*In Vitro*)

The newly synthesized Schiff bases** (L** ^**1**^ **)**–**(L** ^**3**^ **)** and their metal(II) complexes** (1)**–**(12)** have been subjected for the screening of their* in vitro* antibacterial activity against* Escherichia coli*,* Streptococcus faecalis*,* Pseudomonas aeruginosa*,* Klebsiella pneumoniae*,* Staphylococcus aureus,* and* Bacillus subtilis* bacterial strains according to standard procedure [11] and results were reported in Table 1. The obtained results were compared with those of the standard drug streptomycin. The synthesized ligand** (L** ^**1**^ **)** exhibited a significant (16–18 mm) activity against* Streptococcus faecalis*,* Pseudomonas aeruginosa*,* Klebsiella pneumoniae*, and* Bacillus subtilis* bacterial strains and moderate (13-14 mm) activity against* Escherichia coli* and* Staphylococcus aureus*. The ligand** (L** ^**2**^ **)** showed a significant (17-18 mm) activity against* Pseudomonas aeruginosa* and* Staphylococcus aureus,* moderate (13-14 mm) activity against* Escherichia coli*,* Streptococcus faecalis,* and* Bacillus subtilis*, and weaker (10 mm) against* Klebsiella pneumoniae*. The ligand** (L** ^**3**^ **)** demonstrated a significant (16–19 mm) activity against* Escherichia coli* and* Streptococcus faecalis*, moderate (11–15 mm) against* Pseudomonas aeruginosa*,* Klebsiella pneumoniae*, and* Bacillus subtilis,* and weaker (09 mm) activity by* Staphylococcus aureus*. The metal complexes** (4)**,** (5),** and** (8)**–**(10)** displayed overall significant (≥16 mm) activity against all the bacterial strains. Compounds** (1)**–**(3)** exhibited overall a significant (16–20 mm) activity against all bacterial strains except* Streptococcus faecalis* and* Staphylococcus aureus* of** (1)**,* Escherichia coli* and* Klebsiella pneumoniae* of** (2),** and* Staphylococcus aureus* of** (3)** which possessed moderate (12–15 mm) activity. Beside this, the compounds** (6)**,** (7),** and** (9)** exhibited overall a significant (16–24 mm) activity against all bacterial strains except* Streptococcus faecalis* of** (6)** and* Streptococcus faecalis* and* Klebsiella pneumoniae* of** (7)** which possessed moderate (14-15 mm) activity. Also, compound** (11)** showed significant (15–22 mm) activity against* Escherichia coli*,* Streptococcus faecalis*,* Pseudomonas aeruginosa*,* Klebsiella pneumoniae,* and* Staphylococcus aureus*, and moderate (13 mm) activity was shown against* Klebsiella pneumoniae*. Compound** (12)** exhibited significant (15–21 mm) activity against* Escherichia coli*,* Streptococcus faecalis*,* Klebsiella pneumoniae*,* Staphylococcus aureus,* and* Bacillus subtilis*, except* Pseudomonas aeruginosa* which possessed moderate (11–14 mm) activity.

#### 3.8.2. Antifungal Bioassay (*In Vitro*)

The antifungal screening of all compounds was carried out against* Trichophyton mentogrophytes*,* Epidermophyton floccosum*,* Aspergillus niger*,* Microsporum canis*,* Fusarium culmorum,* and* Trichophyton schoenleinii* fungal strains (Table 2) according to the literature protocol [12]. The results of inhibition were compared with the results of standard drugs, miconazole and amphotericin B. The ligand** (L** ^**1**^ **)** possessed significant (57%) activity against* Epidermophyton floccosum* fungal strain, moderate (37–49%) against* Trichophyton mentogrophytes*,* Microsporum canis*,* Fusarium culmorum,* and* Trichophyton schoenleinii*, but no activity against* Aspergillus niger*. The ligand** (L** ^**2**^ **)** showed significant (55–58%) activity against* Trichophyton mentogrophytes* and* Fusarium culmorum* and moderate (39–50%) activity against* Epidermophyton floccosum*,* Aspergillus niger,* and* Trichophyton schoenleinii*, and it was inactive against* Microsporum canis*. However,** (L** ^**3**^ **)** exhibited significant (55–60%) activity against* Fusarium culmorum* and* Aspergillus niger* but showed moderate (38–49%) activity against* Trichophyton mentogrophytes*,* Epidermophyton floccosum*,* Microsporum canis,* and* Trichophyton schoenleinii*. The compound** (1)** showed significant (55–65%) activity against all fungal strains except* Aspergillus niger* strain which had weaker (18%) activity. Similarly, compound** (2)** also possessed significant (55–71%) activity against* Trichophyton mentogrophytes*,* Epidermophyton floccosum*,* Aspergillus niger,* and* Fusarium culmorum* and moderate (41%) activity against* Trichophyton schoenleinii* but weaker (11%) activity against* Microsporum canis*. As well, the compound** (3)** displayed significant (60–68%) activity against* Epidermophyton floccosum* and* Aspergillus niger*, moderate (43–49%) against* Trichophyton mentogrophytes*,* Microsporum canis,* and* Fusarium culmorum,* and also weaker (15%) activity against* Trichophyton schoenleinii*. The compounds** (4)** and** (5)** similarly possessed significant (55–74%) activity against all fungal strains except* Aspergillus niger* strain of compound** (4)** which observed moderate (39%) activity. The compound** (6)** exhibited significant (55–72%) activity against* Trichophyton mentogrophytes*,* Aspergillus niger*,* Microsporum canis*, and* Fusarium culmorum* fungal strains, but strain* Trichophyton schoenleinii* showed moderate (42%) activity and was inactive against* Epidermophyton floccosum*. Besides this, the compound** (7)** demonstrated significant (56–75%) activity against all strains except* Microsporum canis* which had weaker (28%) activity. The compound** (8)** showed significant (56–70%) activity against* Trichophyton mentogrophytes*,* Epidermophyton floccosum*,* Microsporum canis*, and* Trichophyton schoenleinii*, and also moderate (35–42%) activity was observed against* Aspergillus niger* and* Fusarium culmorum*, respectively. The compound** (9)** showed significant (55–69%) activity against* Trichophyton mentogrophytes*,* Microsporum canis*,* Aspergillus niger,* and* Fusarium culmorum and* moderate (38%) activity against* Epidermophyton floccosum* and it was inactive against* Trichophyton schoenleinii*. On the contrary, the compound** (10)** exhibited significant (61–78%) activity against all fungal strains. The compound** (11)** presented significant (55–67%) activity against* Trichophyton mentogrophytes*,* Epidermophyton floccosum*, and* Fusarium culmorum* fungal strains, and other left behind strains* Aspergillus niger*,* Microsporum canis,* and* Trichophyton schoenleinii* showed moderate (36–40%) activity. Similarly, the compound** (12)** showed significant activity (55–70%) against* Epidermophyton floccosum*,* Microsporum canis*, and* Trichophyton schoenleinii* although left behind strains* Trichophyton mentogrophytes*,* Aspergillus niger,* and* Fusarium culmorum* displayed moderate (34–49%) activity. It is obvious from the data reported in Table 2 that** (L** ^**3**^ **)** showed overall good fungal activity as compared to other two ligands. The Ni(II) complex** (10)** of** (L** ^**3**^ **)** was found to be the most active complex. The metal(II) complexes showed enhanced activity results rather than their uncomplexed Schiff bases due to complexation.

#### 3.8.3. Minimum Inhibitory Concentration (MIC)

The synthesized ligands and their transition metal(II) complexes showing promising antibacterial activity (above 80%) were selected for MIC studies and obtained results are reported in Table 3. The antibacterial results indicated that all the metal(II) complexes** (3)**–**(5)** and** (9)**–**(12)** were found to display activity more than 80%; therefore, these complexes were selected for their MIC screening. The MIC values of these compounds fall in the range 32.11 to 53.41 *μ*g/mL. Amongst these, the compound** (12)** was found to be the most active possessing maximum inhibition 32.11 *μ*g/mL against bacterial strain* K. pneumoniae*.

## 4. Conclusions

Three bidentate N, O donor type Schiff bases were prepared by using ethylene-1,2-diamine with 5-methyl-2-furaldehyde, 2-anisaldehyde, and 2-hydroxybenzaldehyde in an equimolar ratio. These ligands were further complexed with transition metals to produce their new metal complexes. Elemental analysis and spectral data of the uncomplexed ligands and their metal(II) complexes were found to be in good agreement with their structures, indicating high purity of all the compounds. All ligands and their metal complexes were screened for antimicrobial activity. The results of antimicrobial activity indicated that metal complexes have significantly higher activity than corresponding ligands. This higher activity might be due to chelation process which reduces the polarity of metal ion by coordinating with ligands.

## Figures and Tables

**Table 1:** Antibacterial bioassay of ligands and their metal(II) complexes (zone of inhibition in mm).

Compounds	(a)	(b)	(c)	(d)	(e)	(f)	SA
(**L** ^1^)	14	18	16	17	13	17	1.94
(**L** ^2^)	13	15	18	10	17	15	2.88
(**L** ^3^)	16	19	14	15	09	14	3.27
(1)	20	14	19	23	16	19	3.15
(2)	15	18	21	14	18	16	2.53
(3)	18	24	18	19	12	16	3.92
(4)	24	21	22	23	21	18	2.07
(5)	26	23	24	23	20	21	2.14
(6)	21	14	17	18	22	16	3.03
(7)	18	15	20	15	19	20	2.32
(8)	17	21	18	19	17	22	2.10
(9)	22	20	19	25	21	22	2.07
(10)	26	22	24	26	23	22	1.83
(11)	22	17	18	12	16	23	4.05
(12)	16	17	15	21	19	17	2.07
**SD**	30	27	28	29	29	28	1.05

(a)* E. coli;  *(b) *S. faecalis;  *(c) *P. aeruginosa;  *(d) *K. pneumoniae;* (e) *S. aureus;  *(f) *B. subtilis*; SD: standard drug; weaker = 0–10 mm, moderate = 11–15 mm, above 15 mm = significant, and SA = statistical analysis.

**Table 2:** Antifungal bioassay of ligands and their metal(II) complexes (% inhibition).

Compounds	(a)	(b)	(c)	(d)	(e)	(f)	SA
(**L** ^1^)	43	57	00	45	48	49	20.33
(**L** ^2^)	58	50	41	00	55	39	21.21
(**L** ^3^)	49	46	55	42	38	60	8.16
(1)	55	65	18	58	56	59	16.94
(2)	65	57	55	11	71	41	21.64
(3)	44	60	68	43	49	15	18.22
(4)	73	62	39	58	67	55	11.71
(5)	60	71	78	66	57	74	8.16
(6)	61	00	63	55	67	42	25.07
(7)	63	72	59	28	56	68	15.65
(8)	58	70	35	56	42	63	13.13
(9)	69	38	56	60	55	00	24.84
(10)	63	72	78	75	61	70	6.68
(11)	56	67	38	40	55	36	12.48
(12)	43	55	34	70	49	62	13.01

(a) *T. mentogrophytes;* (b) *E. floccosum;* (c) *A. niger;* (d) *M. canis;* (e) *F.culmorum;* (f) *T. schoenleinii*; weaker = 0–30%, moderate = 31–54%, 55–100% = significant, and SA = statistical analysis.

**Table 3:** Minimum inhibitory concentration (*μ*g/mL) of the selected compounds (3)–(5) and (9)–(12) against selected bacteria.

Number	*E. coli *	*S. faecalis *	*P. aeruginosa *	*K. pneumoniae *	*S. aureus *	*B. subtilis *
(3)	—	52.64	—	—	—	—
(4)	45.68	—	—	—	—	—
(5)	52.17	33.16	35.34	—	—	—
(9)	—	—	—	51.22	—	—
(10)	38.34	47.21	44.41	33.67	—	—
(11)	—	—	—	—	—	49.26
(12)	53.41	35.67	43.94	32.11	40.33	47.82
